# Dipterans Associated with a Decomposing Animal Carcass in a Rainforest Fragment in Brazil: Notes on the Early Arrival and Colonization by Necrophagous Species

**DOI:** 10.1673/031.013.14501

**Published:** 2013-12-07

**Authors:** Simao D. Vasconcelos, Tadeu M. Cruz, Roberta L. Salgado, Patricia J. Thyssen

**Affiliations:** 1 Necrophagous Insects Research Group, Department of Zoology, Universidade Federal de Pernambuco, Recife, Pernambuco, Brazil; 2 Present address: Department of Scientific Police, Government of Pernambuco State, Recife, Pernambuco, Brazil; 3 Department of Microbiology and Parasitology, Universidade Federal de Pelotas, Capao do Leao, Rio Grande do Sul, Brazil

**Keywords:** blow flies, flesh flies, forensic science, forensic entomology, homicide, rainforest

## Abstract

This study aimed to provide the first checklist of forensically-important dipteran species in a rainforest environment in Northeastern Brazil, a region exposed to high rates of homicides. Using a decomposing pig, *Sus scrofa* L. (Artiodactyla: Suidae), carcass as a model, adult flies were collected immediately after death and in the early stages of carcass decomposition. To confirm actual colonization of the carcass, insects that completed their larval development on the resource were also collected and reared until adult stage. A diverse assemblage of dipterans composed of at least 28 species from seven families with necrophagous habits was observed within minutes after death. Besides Calliphoridae and Sarcophagidae, species from forensically-important families such as Phoridae, Anthomyiidae, and Fanniidae were also registered. Eleven species were shown to complete their development on the carcass. The majority of individuals emerged from larvae collected at the dry stage of decomposition. *Hemilucilia segmentaria* Fabricius (Diptera: Calliphoridae), *H. semidiaphana* (Rondani), and *Ophyra chalcogaster* (Wiedemann) (Muscidae) were the dominant species among the colonizers, which supports their importance as forensic evidence in Brazil.

## Introduction

The temporal pattern of arrival of necrophagous insects at a cadaver is a key feature in the estimation of the minimum post-mortem interval, which is the most widespread contribution of forensic entomology. Information on abiotic factors combined with the time interval taken by the larvae to reach each developmental stage can provide reliable estimates of the time elapsed between cadaver colonization by insects and the discovery of the body (Catts and Goff 1992; Benecke 1998; Grassberger et al. 2003; Tomberlin et al. 2011).

In forensic studies, decomposition is divided into stages, the number and duration of which vary according to the region, climate, and other environmental factors. The changes in a cadaver that occur immediately following death are often more rapid than those that take place later during the decomposition (Goff 2010). Therefore, in order to validate entomological evidence related to the period of insect activity, shorter time scales in field surveys of necrophagous insects are likely to increase the reliability of the estimates. Additionally, it is crucial to understand the dynamics of cadaver detection and colonization as soon as death occurs.

It is a widely accepted assumption that dipteran species of the families Sarcophagidae (flesh flies) and Calliphoridae (blow flies) are able to reach cadavers within a few hours of death and are the first colonizers of a corpse (Bornemissza 1957; Payne 1965; Hall and Doisy 1993; Anderson and Van Laerhoven 1996; Tabor et al. 2004, 2005). This ability has led to the more frequent use of sarcophagids and calliphorids as evidence in medico-criminal investigations (Catts and Goff 1992). However, references to the early arrival of necrophagous dipterans on a cadaver frequently seem to overlook species of other families, such as Piophilidae, Anthomyiidae, and Fanniidae. Moreover, field surveys based solely on the collection of adults may fail to detect whether the species actually colonize the corpse as a resource for larval development (Oliveira and Vasconcelos 2010).

The development of forensic entomology in Brazil has been sustained by an increasing number of field surveys of necrophagous species, comprising ecosystems located mainly in the Amazon and in central and southern states of the country. Areas with high rates of homicides, such as cities located in the Northeastern region, have been neglected (Vasconcelos and Araujo 2012). In this context, this study aimed at providing a preliminary checklist of forensically important dipteran species in a rainforest fragment in Northeastern Brazil. Two hypotheses were tested: 1) species of Calliphoridae and Sarcophagidae would be the first insects to locate a recently killed animal, and 2) larval competition during colonization would favor a limited number of species that would be able to complete their cycle on the carcass. To test these hypotheses, a pig carcass was used as a model to investigate which species would actually colonize the ephemeral resource, as compared to species that would be mostly limited to visiting the resource as adults.

The study was carried out in Recife, one of Brazil's largest cities (population 3.7 million), located on the Northeastern coast. It ranks among the most violent cities in the country, with a rate of 57.9 homicides/100,000 inhabitants, and many of the homicides are unsolved (Waiselfisz 2011).

## Materials and Methods

The field study took place in a preserved rainforest fragment (Dois Irmaos State Park) in Recife (08° 07′ S; 34° 52′ W). The park has a total area of 388 ha, with an altitude ranging from 30 to 80 m a.s.l. The local climate is hot and humid, with mean rainfall ca. 2,500 mm/year, an average annual temperature ca. 25.6° C, and two well-defined seasons, namely dry (October-February) and rainy (March-September). Vegetation is classified as dense, ombrophylous forest composed mainly of Fabaceae, Lauraceae, Moraceae, Sapotaceae, and Euphorbiaceae species (Machado et al. 1998). The area was chosen because it has been used as a repository for the clandestine dumping of cadavers from homicides.

A pig, *Sus scrofa* L. (Artiodactyla: Suidae) (ca. 15 kg) was used as the model. The pig was killed *in loco* with a gunshot to the occipital region, a procedure performed by experts according to the Ethics Committee of the Federal University of Pernambuco. Immediately after death, the carcass was placed in a metal cage (0.9 m × 0.6 m × 0.5 m) to prevent disturbance by large scavengers. Around the cage, a metal frame (2 m high × 1 m long × 1 m wide) covered with a fine white mesh fabric was placed in order to trap insects that visited the carcass. A 30 cm gap between the bottom of the net and the soil was left, through which insects could gain access to the carcass. The field experiment took place in July 2007, in the rainy season. The average temperature throughout the experiment was 25.2° C, and the mean relative humidity was 84%.

Death occurred on day 1 at 13:00. For the collection of early species, samples were taken at seven timepoints, which combined are referred to hereafter as “immediately postdeath”: 5, 30, 60, 90, 120, 150, and 180 min post-mortem. At each of these timepoints, the adult flies trapped in the mesh structure were collected using an entomological net (20 cm diameter), sweeping for 5 min each time To determine which species would continue to visit the resource, an additional collection of dipteran adults on the carcass was performed at 24, 48, and 72 hr postmortem using the same procedure. Collected insects were killed using ethyl acetate, mounted, and identified using taxonomical keys (Lopes 1946; McAlpine et al. 1981, 1987; Dear 1985; Carvalho et al. 2002; Mello 2003; Carvalho and MelloPatiu 2008). All specimens were deposited at the Entomological Collection at the Universidade Federal de Pernambuco, Brazil.

In order to collect larvae at the post-feeding stage, i.e., insects that completed the larval stage on the carcass but were yet to pupate, a 60 cm × 30 cm × 15 cm plastic tray containing sawdust was placed under the cage, onto which insects would fall, as they typically pupate on the soil. Starting on the fourth day postmortem, the tray was removed daily until the 11^th^ day, from which point the tray was retrieved every 48 hr until the 17^th^ day post-mortem. This was due to previous observational studies that indicated that the majority of pupation occurred in that time interval .

All immature insects recovered from the tray on each day were placed in plastic containers (31 cm × 18 cm × 10 cm) covered with fine nylon mesh and containing a Petri dish with ca. 20 g of minced beef to guarantee that the larvae completed their development cycle. Rearing conditions in the glasshouse emulated field conditions (mean temperature: 27.8 ± 1.6° C; RH: 61.6 ± 9.8%; 12:12 L:D photoperiod). Insects were observed daily, and emerged adults were identified to the lowest taxonomie level. The frequency of occurrence of each species at each decomposition stage was calculated. Chi-square tests using the significance level of 5% were performed to check for differences in the abundance of necrophagous species according to the stage of decomposition.

## Results

### Insect species as early visitors

In total, 153 insects from 14 families were collected in the first three hours after death (Table 1). This included species of Phoridae (24.2% of all adults), Sarcophagidae (18.3%), Piophilidae (10.5%), Calliphoridae (10.5%), Fanniidae (8.5%), Chloropidae (6.5%), Muscidae (4.6%), Dixidae (4.6%), and, in smaller proportions, Milichiidae, Drosophilidae, Anthomyiidae, Micropezidae, Ropalomeridae, and Neriidae. Sarcophagidae was the richest family, with eleven species, most of which belonged to the genus *Oxysarcodexia* (Table 1). Other necrophagous families were represented by fewer species, as Anthomyiidae had 3 species, and Calliphoridae, Muscidae, Fanniidae, Phoridae, Piophilidae, each had two species. *Megaselia scalaris* Loew (Phoridae) was the most abundant species (19.6% of all specimens) at the period immediately after death. Only nine species with no previous record of necrophagy were registered in this time interval (Table 1).

**Figure 1.**
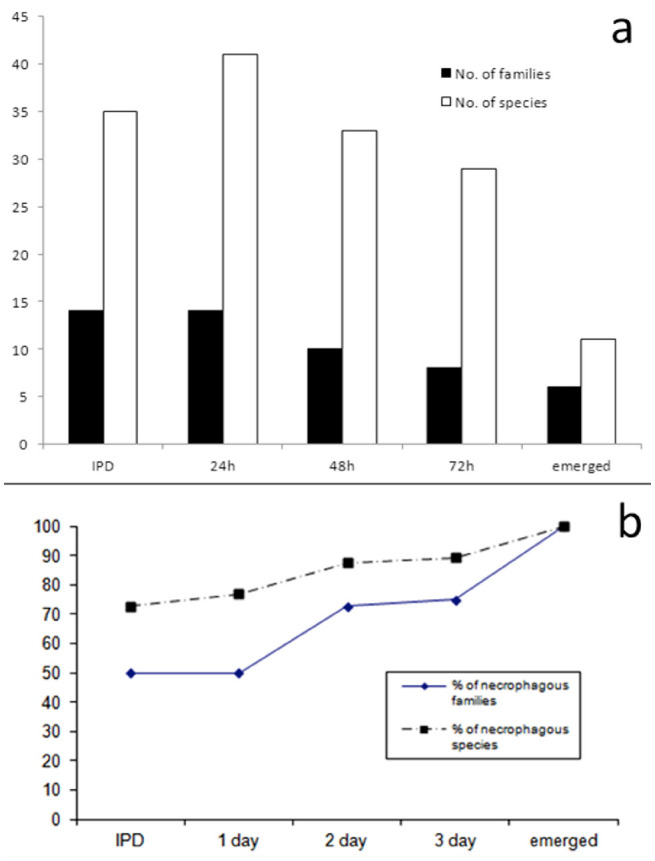
Diptera diversity in a decomposing carcass at different intervals postmortem: (a) absolute number of families and species registered on the carcass, and (b) percentage of families and species with a previous record of a necrophagous habit. IPD = immediately post-death. High quality figures are available online.

Twenty five species from 12 families were collected within the first 30 minutes of death (Table 1). In fact, 16 species were collected as early as five minutes post-death: *Fly str i - cocnema plinthopyga* (Wiedmann) (Sarcophagidae), *Oxysarcodexia modesta* Lopes, *O. fluminensis* Lopes, *(O. riograndensis* Lopes, *O. intona* (Curran and Walley), *O*. *avuncular* (Lopes), *O. excise* (Lopes), and *Peckia (Squamalodes) in gen s* (Walker); *Hemilucilia segmentaria* (F.) (Calliphoridae), *H. semidiaphana* (Rondani); *Morellia humeralis* (Stein) (Muscidae); *Piophila casei* L. (Piophilidae), Piophilidae sp.; *Fannia obscurinervis* (Stein) (Fanniidae), *Fannia* sp.l; and *Anthomyia punctipennis* (Wiedemann) (Anthomyiidae). From that moment on, necrophagous species continued to visit the carcass for at least 72 hr post-death, while the diversity of non-necrophagous (predatory, accidental, and omnivore species) diminished throughout time (Table 1). To illustrate that, at three days post-death the number of families and species associated with the carcass was reduced by 42.9% and 15.2% respectively when compared to immediately post-death (Figure 1). The frequency of necrophagy of the insect species registered on the carcass increased throughout decomposition, as the percentage of necrophagous species rose from 72.7% immediately post-death to 89.3% at 72 hr post-death (Figure 1).

**Table 1.**
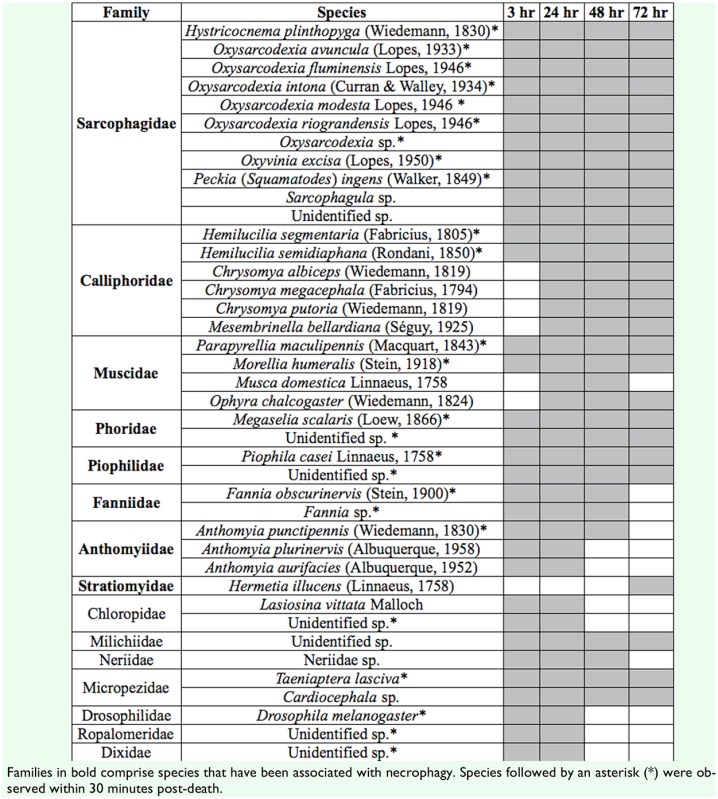
Occurrence (shaded cells) of dipteran insects associated with a decomposing carcass in a rainforest fragment in Northeast Brazil at different time intervals after death.

### Insect species as colonizers

A total of 18,469 adults emerged from the samples collected at the post-feeding stage. Adults began to emerge from the fourth day post-death (when the carcass was at the bloated stage) until skeletonization of the carcass, which occurred on the 17^th^ day. Adults from 11 species belonging to six families emerged; the majority of individuals (61.6% of all adults) corresponded to Calliphoridae, followed by specimens from Phoridae (25.6%) and Muscidae (11.6% of the emerged adults) (Table 2). Two Calliphoridae species were dominant in terms of abundance: *H. segmentaria* (34.4% of emerged adults) and *H. semidiaphana* (27.2%). *Ophyra chalcogaster* (Wiedemann) (Muscidae) and *M. scalaris* (Phoridae) also composed a significant proportion of the emerged adults.

**Table 2.**
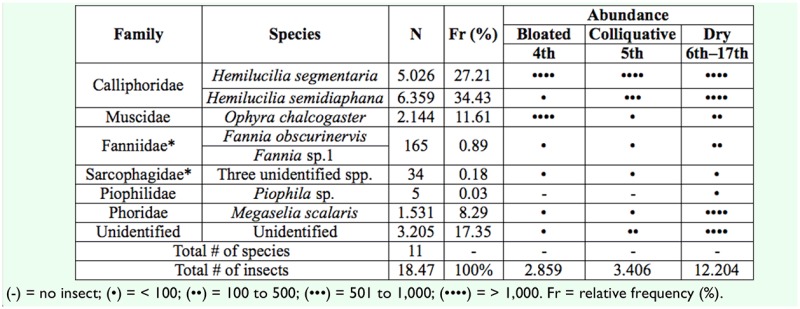
Species that completed their larval development on the carcass according to their abundance and stage of decomposition (days post-death) at which larvae were retrieved.

Decomposition occurred quickly due to a combination of biotic and abiotic factors, which included the action of maggots, whose population reached thousands of individuals, and environmental factors such as rainfall and elevated temperature. The stages of decomposition were characterized as follows: fresh stage (048 hr post-death), bloated (48–96 hr), decay (96–120 hr), and dry stage (120-ca. 410 hr post-death). After that period, virtually no insects were found on the carcass. The amount of emerged adults differed according to the decomposition stage at which larvae were recovered: 15.5% of the adults emerged from larvae collected at the bloated stage, 18.4% at the decay stage, and 66.1% of the adults emerged from larvae collected at the dry stage, and this difference was statistically significant (χ^2^ = 5,757; *p <* 0.0001, df = 3). The diversity of emerged adults differed little according to the stage in which the larvae were retrieved, with the exception of *Piophila* sp., whose larvae were only collected at the dry stage. The temporal pattern of emergence varied. While the majority of *H. semidiaphana* and *M. scalaris* adults emerged when larvae were collected at dry stage, the numbers of *O. chalcogaster* larvae retrieved decreased with decomposition (Table 2). Regarding the larvae reared in the laboratory, the minimum time of emergence of adults was as short as four days after collection for *H. semidiaphana* and *H. segmentaria* and as long as 14 days for *M. scalaris.*

## Discussion

When conducting field surveys on necrophagous species on animal carcasses, the first hours post-death are critically important for the establishment of dipteran populations, as not all species continue to explore the cadaver throughout its decomposition. The presence of several non-necrophagous species at early stages post-death confirms the notion that a corpse is exploited not only by necrophagous species, but by herbivore, predatory, and omnivore species that are attracted by the necrophagous fauna or exploit the resource as a complementary source of food or as a temporary habitat (Smith 1986; Braack 1987). The diversity of feeding habits in insect assemblages has been consistently found in other field studies performed in several countries such as Brazil (Carvalho and Linhares 2001), the United States (Anderson and Van Laerhoven 1996), South Africa (Braack 1987), Spain (Baz et al. 2010), and Colombia (Segura et al. 2011).

The amount of time after death affects the structure of the assemblage of insects attracted to a carcass (Hall and Doisy 1993), a feature that will have direct implications on the accuracy of the biological information available to the forensic entomologist. In this study, species from seven forensically important families with varying degrees of specialization in necrophagy were recorded minutes after death: Calliphoridae, Muscidae, Sarcophagidae, Phoridae, Piophilidae, Anthomyiidae, and Fanniidae. Numerous references endorse Calliphoridae and Sarcophagidae as the first arthropods to locate and colonize a cadaver (Payne 1965; Hall and Doisy 1993; Anderson and Van Laerhoven 1996; Tabor et al. 2004, 2005). For example, Reibe and Madea (2010) reported that egg batches of *Lucilia cesar* (Calliphoridae) were detected on the carcass just two hours after its exposure in the field.

The data presented here confirm the ability of calliphorids and sarcophagids to quickly locate dead animal matter, but reveal that *M. scalaris* (Phoridae), *P. casei* (Piophilidae), *F. obscurinervis* (Fanniidae), and *M. humeralis* (Muscidae), among others, can reach the carcass as quickly as five minutes after death. This is, to our knowledge, the documentation of the earliest arrival of these and other species (Table 1) on a carcass based on a field experimental setting. Piophilidae, for example, has been largely associated with the late stages post-death and are commonly found in both urban and rural environments (Martin-Vega 2011). Although the forensic relevance of insect species has been largely related to the recovery of larvae from the corpse, the presence of adult phorids and piophilids as forensic evidence at fresh and bloated stages of decomposition should not be dismissed.

While the forensic relevance has been corroborated for some of the early species registered here, namely *Chrysomya* species (Grassberger et al. 2003), *M. scalaris* (Greenberg and Wells 1998), *P. casei* (Benecke 1998), and *Fannia* species (Benecke and Lessig 2001), species from the genus *Oxysarcodexia* are comparatively less studied. The genus is characteristic of the Neotropical region, and the greatest number of species is found in Brazil, where they develop preferentially in feces (Lopes 1946). Recently, *O. riograndensis* was found colonizing cadavers at the Institute of Legal Medicine in Recife (Oliveira and Vasconcelos 2010), which encourages further studies to assess their forensic importance. The arrival of the black soldier-fly, *Hermetia illucens* (Stratiomyidae), at later stages of decomposition was previously demonstrated by Pujol - Luz et al. (2008), who calculated the time of development of larvae to estimate the time of death in a criminal case in Brazil.

Perhaps the best way to validate the forensic relevance of an insect species is to assess whether it can effectively complete its larval cycle using the corpse as substrate. In the animal model in our study, an initial assemblage composed of species with diverse feeding habits changed into a more necrophagy-oriented community. This was evident from the first days post-death and, naturally, reached a maximum specialization when species collected at the post-feeding stage were taken into consideration. Only a third of the necrophagous species collected as adults effectively completed the cycle to adult stage. It is likely that several fly species began their development on the carcass, but direct effects of interspecific competition resulted in a lower number of species being able to successfully complete their development on the resource. Physiological, morphological, and behavioral characteristics of larvae of different species will determine strategies for resource exploitation, which in turn will generate different patterns in the emerged populations (Denno and Cothran 1976). Even considering that the collections were based on a single carcass, the high number of colonizing species (11) in the forest fragment located in an urban area may be of use in its extrapolation to human cadavers, as pigs have been systematically considered to be the best animal models to mimic human decomposition in forensic entomology studies (Catts and Goff 1992).

Three families stood out in terms of constancy and abundance: Calliphoridae, Muscidae, and Phoridae. Two Calliphoridae species, *H. segmentaria* and *H. semidiaphana*, besides having been recorded immediately post-death, came out as the dominant emerged adults. The genus *Hemilucilia* comprises six species distributed in several countries in Central and South America, four of which are found in Brazil, especially in forested areas (Linhares 1981). Surveys performed in southern Brazil (Carvalho et al. 2000) demonstrated that their abundance and intimate association with human cadavers encourages forensic entomologists to consider them as candidates for use in medico-legal investigations. Surprisingly, no adults from *Chrysomya* species emerged from the carcass despite numerous references of their escalating distribution in Northeast Brazil (Vasconcelos and Araujo 2012) and the recent register of *C. megacephala* on human cadavers in Recife (Oliveira and Vasconcelos 2010). This could be a result of direct competition with native *Hemilucilia* species, which still seem to be more successful in locating and colonizing carcasses in forested environments.

*Opyhra chalcogaster* was a dominant species found mostly at early stages of decomposition. *Ophyra* species (Muscidae) have been associated with both cadavers (Carvalho et al. 2000) and carcasses (Tantawi et al. 1996), especially during active decay stages. Phoridae species were also among the most abundant emerged adults, although a recent study performed in Malaysia led to the conclusion that species from this family tend to be dominant when corpses are located indoors (Kumara et al. 2012).

Despite the richness of species reported throughout the first days post-death, Sarcophagidae was classified as an accessory group, as they represented only 0.18% of all emerged adults. The high diversity associated with low abundance of Sarcophagidae observed in the rainforest fragment of Dois Irmaos State Park has also been found in other field studies (Mulieri et al. 2008). The other dominant families among the emerged adults, Muscidae and Phoridae, are also commonly reported in larval stages in field experiments on forensic entomology (Segura et al. 2011).

In tropical regions with high rates of unsolved homicide, such as the case of Northeast Brazil, forensic scientists should be aware of the fact that decomposition-related processes occur at a fast rate, increasing the difficulty in establishing definite chronological stages and, consequently, the insect community associated with them. This reinforces the necessity of an immediate involvement of the forensic scientist in search for entomological evidence, preferably at larval stage, because a shorter window for data gathering is available.

Despite the limitations of using a single carcass as model, due to logistical and ethical restraints, this study provides the first evidence of at least 10 species completing their larval cycle on carrion in rainforest fragments in Northeastern Brazil, which include *H. segmentaria, H. semidiaphana, O. chalcogaster, F. obscurinervis,* and *M. scalaris.* The Neotropical *Hemilucilia* species in particular deserve further studies as useful forensic indicators, especially considering their recent use in the estimation of minimum post-mortem interval in Brazil (Kosmann et al. 2011). Because of the overlap in the temporal occupation of some Diptera species, only detailed bionomical studies can lend support to their use as reliable indicators of the period of insect activity on the corpse. Finally, the common assumption that Sarcophagidae and Calliphoridae are the sole visitors at early stages post-death should be regarded with caution.

## References

[bibr01] Anderson GS, Van Laerhoven SL. (1996). Initial studies on insect succession on carrion in southwestern British Columbia.. *Journal of Forensic Sciences*.

[bibr02] Baz A, Cifriani B, Martin-Vega D, Baena M. (2010). Phytophagous insects captured in carrion-baited traps in central Spain.. *Bulletin of Insectology*.

[bibr03] Benecke M. (1998). Six forensic entomology cases: description and commentary.. *Journal of Forensic Sciences*.

[bibr04] Benecke M, Lessig R. (2001). Child neglect and forensic entomology.. *Forensic Science International*.

[bibr05] Bornemissza GF. (1957). Analysis of arthropod succession in carrion and the effect of its decomposition on the soil fauna.. *Australian Journal of Zoology*.

[bibr06] Braack L. (1987). Community dynamics of carrion-attendant arthropods in tropical African woodland.. *Oecologia*.

[bibr07] Carvalho CJB, Mello-Patiu CA. (2008). Key to the adults of the most common forensic species of Diptera in South America.. *Revista Brasileira de Entomologia*.

[bibr08] Carvalho CJB, Moura MO, Ribeiro PB. (2002). Chave para adultos de dípteros (Muscidae, Fanniidae, Anthomyiidae) associados ao ambiente humano no Brasil.. *Revista Brasileira de Entomologia*.

[bibr09] Carvalho LML, Linhares AX. (2001). Seasonality of insect succession and pig carcass decomposition in a natural forest area in Southeastern Brazil.. *Journal of Forensic Sciences*.

[bibr10] Carvalho LML, Thyssen PJ, Linhares AX, Palhares FAB. (2000). A checklist of arthropods associated with pig carrion and human corpses in southeastern Brazil.. *Memorias do Instituto Oswaldo Cruz*.

[bibr11] Catts EP, Goff ML. (1992). Forensic entomology in criminal investigations.. *Annual Review of Entomology*.

[bibr12] Dear JP. (1985). A revision of the New World Chrysomyini (Diptera: Calliphoridae).. *Revista Brasileira de Zoología*.

[bibr13] Denno RF, Cothran WR. (1976). Competitive interactions and ecological strategies of Sarcophagid and Calliphorid flies inhabiting rabbit carrion.. *Annals of the Entomological Society of America*.

[bibr14] Goff ML.., Amendt J, Goff ML, Campobasso CP, Grassberger M (2010). Early post-mortem changes and stages of decomposition.. *Current Concepts in Forensic Entomology*.

[bibr15] Grassberger M, Friedrich E, Reiter C. (2003). The blowfly *Chrysomya albiceps* (Wiedemann) (Diptera: Calliphoridae) as a new forensic indicator in Central Europe.. *International Journal of Legal Medicine*.

[bibr16] Greenberg B, Wells JD. (1998). Forensic use of *Megaselia abdita* and *M. scalaris* (Phoridae: Diptera): case studies, development rates, and egg structure.. *Journal of Medical Entomology.*.

[bibr17] Hall RD, Doisy KE. (1993). Length of time after death: effect on attraction and oviposition or larviposition of midsummer blow flies (Diptera: Calliphoridae) and flesh flies (Diptera: Sarcophagidae) of medicolegal importance in Missouri.. *Annals of the Entomological Society of America*.

[bibr18] Kosmann C, Macedo MP, Barbosa TAF, Puj ol-Luz JR. (2011). *Chrysomya albiceps* (Wiedemann) and *Hemilucilia segmentaria* (Fabricius) (Diptera, Calliphoridae) used to estimate the postmortem interval in a forensic case in Minas Gérais, Brazil.. *Revista Brasileira de Entomología*.

[bibr19] Kumara TK, Disney RHL, Abu Hassan A, Flores M, Tan Siew H, Zulqarnain M, CheSalmah MR, Bhupinder S. (2012). Occurrence of oriental flies associated with indoor and outdoor human remains in the tropical climate of north Malaysia.. *Journal of Vector Ecology*.

[bibr20] Linhares AX. (1981). Synanthropy of Calliphoridae and Sarcophagidae (Diptera) in the city of Campinas, Sao Paulo, Brazil.. *Revista Brasileira de Entomología*.

[bibr21] Lopes HS. (1946). Contribuiçào ao conhecimento das espécies do género Oxysarcodexia Townsend, 1917 (Diptera, Sarcophagidae).. *Boletim da Escola Nacional de Veterinária*.

[bibr22] Machado IC, Lopes AV, Porto KC. (1998). *Dois Irmãos State Park: Studies in a Rainforest Remnant in an Urban Area (Recife, Pernambuco, Brazil).*.

[bibr23] Martin-Vega D. (2011). Skipping clues: Forensic importance of the family Piophilidae.. *Forensic Science International*.

[bibr24] McAlpine JF, Peterson BV, Shewell GE, Teskey HJ, Vockeroth JR, Wood DM. (1981). *Manual of Neartic Diptera*.

[bibr25] McAlpine JF, Peterson BV, Shewell GE, Teskey HJ, Vockeroth JR, Wood DM. (1987). *Manual of Neartic Diptera*.

[bibr26] Mello RP. (2003). Key to the identification of adult stages of species of families Calliphoridae (Diptera, Brachycera, Cyclorrhapha) found in Brazil.. *Entomología y Vectores*.

[bibr27] Mulieri PR, Schnack JA, Mariluis JC, Torretta JP. (2008). Flesh flies species (Diptera: Sarcophagidae) from a grassland and a woodland in a Nature Reserve of Buenos Aires, Argentina.. *Revista de Biología Tropical*.

[bibr28] Oliveira TC, Vasconcelos SD. (2010). Insects (Diptera) associated with cadavers at the Institute of Legal Medicine in Pernambuco, Brazil and its implications for forensic entomology.. *Forensic Science International*.

[bibr29] Payne JA. (1965). A summer carrion study of the baby pig *Sus scrofa* Linnaeus.. *Ecology*.

[bibr30] Pujol-Luz JR, Francez PAC, Ururahy-Rodrigues A, Constantino R. (2008). The black soldier-fly, *Hermetia illucens* (Diptera, Stratiomyidae), used to estimate the postmortem interval in a case in Amapá state, Brazil.. *Journal of Forensic Sciences*.

[bibr31] Reibe S, Madea B. (2010). How promptly do blowflies colonise fresh carcasses? A study comparing indoor with outdoor locations.. *Forensic Science International*.

[bibr32] Segura NA, Bonilla MA, Usaquen W, Bello F. (2011). Entomofauna resource distribution associated with pig cadavers in Bogota DC.. *Medical and Veterinary Entomology*.

[bibr33] Smith KGV. (1986). *A Manual of Forensic Entomology.*.

[bibr34] Tabor KL, Brewster CC, Fell RD. (2004). Analysis of the successional patterns of insects on carrion in southwest Virginia.. *Journal of Medical Entomology*.

[bibr35] Tabor KL, Fell RD, Brewster CC. (2005). Insect fauna visiting carrion in southwest Virginia.. *Forensic Science International*.

[bibr36] Tantawi TI, El-Kady EM, Greenberg B, El-Chaffar HA. (1996). Arthropod succession on exposed rabbit carrion in Alexandria, Egypt.. *Journal of Medical Entomology*.

[bibr37] Tomberlin JK, Mohr R, Benbow ME, Tarone AM, VanLaerhoven S. (2011). A roadmap for bridging basic and applied research in forensic entomology.. *Annual Review of Entomology*.

[bibr38] Vasconcelos SD, Araujo MSC. (2012). Necrophagous species of Diptera and Coleoptera in Northeastern Brazil: State of the art and challenges for the forensic entomologist.. *Revista Brasileira de Entomología*.

[bibr39] Waiselfisz JJ. (2011). *O Mapa da Violencia no Brasil - Os Novos Padroes da Violencia Homicida no Brasil.*.

